# Behavior of Water-to-Air Trace Gas Exchange for Ammonia and Amines at Low Wind Speeds Using Chemical Ionization Mass Spectrometry

**DOI:** 10.1021/acsearthspacechem.6c00080

**Published:** 2026-07-30

**Authors:** Christine Troller, Stephen D. Archer, Coty N. Jen

**Affiliations:** † Department of Chemical Engineering, 6612 Carnegie Mellon University, 5000 Forbes Ave, Pittsburgh, Pennsylvania 15213, United States; ‡ Center for Atmospheric Particle Studies, 6612 Carnegie Mellon University, 5000 Forbes Ave, Pittsburgh, Pennsylvania 15213, United States; § Bigelow Laboratory for Ocean Sciences, 60 Bigelow Drive, East Boothbay, Maine 04544, United States

**Keywords:** Ammonia, alkylamines, trace gas exchange, low wind speed fluxes, chemical ionization mass spectrometry

## Abstract

Ammonia (NH_3_) and alkylamines play critical roles in atmospheric aerosol chemistry, influencing cloud formation, Earth’s radiative balance, and air quality. Despite significant contributions from water-based sources, associated emissions of ammonia and alkylamines remain poorly characterized, especially in freshwater environments. As such, this lack of understanding complicates efforts to accurately predict ammonia and amine fluxes from aquatic sources. For example, the widely used two-film resistance model often leads to substantial misestimation of these fluxes under low-wind conditions. Compounding this uncertainty, Henry’s Law, which is commonly applied to these compounds, relies on published Henry’s Law constants that can differ by more than 2 orders of magnitude, further resulting in inaccurate modeling of the water-to-air transfer of these highly polar gases. In this study, we experimentally investigate the real-time trace gas exchange (i.e., flux) of ammonia and alkylamines in model aqueous solutions (10^–9^ to 10^–5^ M) across the air–water interface using a custom flux measurement system (FMS) coupled with a hydronium chemical ionization mass spectrometer (CIMS). Carrier gas velocities ranged from 10^–4^ to 10^–3^ m s^–1^ to examine how low wind speeds influence emission rates. Observed gas concentrations within the FMS headspace were compared to theoretical concentrations predicted by the two-film resistance model and Henry’s Law. Our findings provide direct experimental evidence of ammonia and alkylamine gas exchange from aqueous systems in low wind conditions, helping to constrain their emission behavior and refine our understanding of aquatic contributions to the atmosphere.

## Graphical abstract

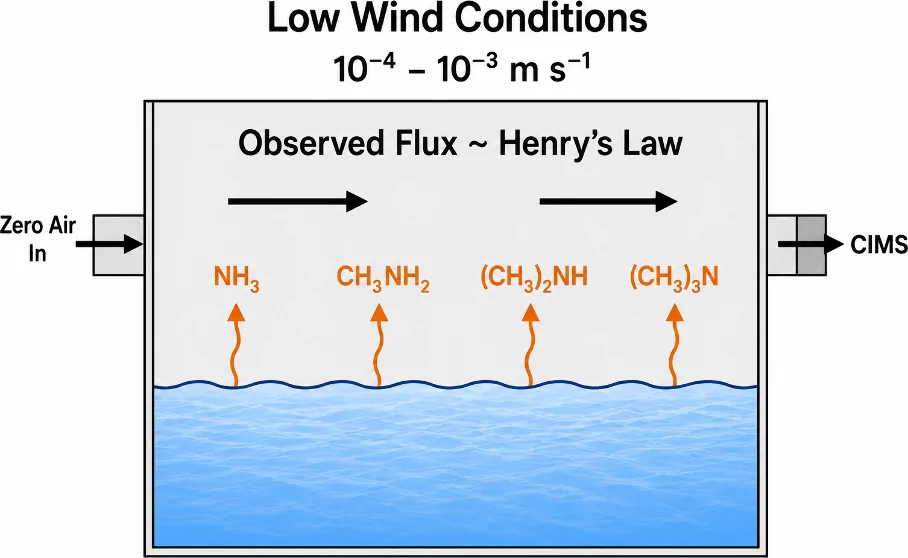

## Introduction

Ammonia (NH_3_) and alkylamines play a crucial role in atmospheric aerosol chemistry and climate. Along with participating in Earth’s nitrogen cycle, these gaseous bases have also been shown to react with sulfuric acid vapor and nucleate to form atmospheric aerosol particles. Higher concentrations of atmospheric particles have proven to influence cloud properties and ultimately, Earth’s radiative balance.^1,2^ Previous studies reported that even low concentrations of these basic compounds (i.e., a few parts per trillion by volume (pptv) or ∼ 10^7^ cm^–3^), can increase nucleation rates of atmospheric particles by orders of magnitude.^1–4^ Furthermore, ammonia and alkylamines can degrade air quality. Reactions involving hydroxyl radicals and amines can form toxic compounds, such as nitrosamines and nitramines.^5,6^ In addition, ammonia, and to a lesser extent alkylamines, contributes to elevated fine particulate matter mass, which poses serious health risks.^7,8^ As such, advancing experimental understanding of how ammonia and alkylamines are emitted to the atmosphere is essential for accurately representing their atmospheric abundance and associated climate and health impacts.

Ammonia and alkylamines are widespread in the environment, produced by both natural and anthropogenic sources (e.g., decomposition of organic matter, agriculture, industrial activities, biological processes). Agriculture and biomass burning are thought to be the dominant atmospheric sources of ammonia and alkylamines on land and are estimated to contribute about 50000 GgN yr^–1^ ammonia and 200 GgN yr^–1^ aliphatic amines.^8–13^ However, water sources of ammonia and alkylamines have not been fully characterized and require investigation to determine their contribution to atmospheric abundances. Significant natural generation and consumption of ammonia and alkylamines in oceans and lakes originate from biological activity involving phytoplankton and their surrounding microbial community,^14,15^ as well as sedimentary reservoirs, where these compounds can absorb to particles and be rapidly released to the water column during sediment resuspension events.^16^ Amines can either be directly emitted from aquatic microorganisms or formed through the degradation of aquatic organic matter involving complex pathways.^15^ Once produced in surface waters, these compounds may be transferred to the atmosphere through air–water exchange, where they can influence atmospheric composition and particle formation. Despite their presumed importance to atmospheric processes over water,^14,17,18^ the mechanisms and rates governing ammonia and alkylamine transfer across air–water interfaces remain poorly constrained by direct, experimental measurements.

Gas concentrations of ammonia and alkylamines in the atmosphere are directly dependent on their aqueous concentration and water-to-air exchange rate (i.e., flux) into the atmosphere. In water, the highly water-soluble ammonia and alkylamines mainly exist in their cation form and account for the majority of the total dissolved concentration.^15^ Previously reported natural liquid concentrations of ammonia and amines range from 10^–9^ to 10^–7^ M of the combined protonated and unprotonated compounds.^19–23^ However, water-to-air exchange only occurs with the volatile unprotonated form, providing a source of gaseous ammonia and alkylamines to the atmosphere.^15^ A number of studies have examined ammonia fluxes from the ocean.^14,24–26^ However, even fewer studies of waters from the tropical Atlantic Ocean region and Arabian Sea have reported alkylamine fluxes, measured via offline analysis.^14,15^ In such methods, samples are collected and analyzed after the experiment, limiting temporal resolution and the ability to capture dynamic emission behavior. In contrast, online measurements enable real-time, continuous detection and allows for direct quantification of temporal variability and air–water fluxes. No studies have reported online measurements of alkylamines from marine environments, while only one study has reported online alkylamine fluxes from freshwater lake samples.^27^ Furthermore, a few studies have investigated total liquid alkylamine concentrations in seawater,^14,19,21–23^ but there are very few known measurements of amines in freshwater environments, such as lakes, rivers, and reservoirs.^28^ As a result, flux parametrizations for alkylamines are poorly constrained, limiting our confidence in model-based estimates of their role in atmospheric chemistry over water.

In the absence of online flux measurements, water-to-air exchange of soluble trace gases is typically represented using resistance-based models, most commonly the two-film resistance model.^29^ This framework conceptualizes gas transfer as occurring across thin air-side and water-side boundary layers and enables estimation of fluxes from bulk water and atmospheric concentrations. Its relative simplicity and computational efficiency make it particularly well suited for regional and global atmospheric models, which require parametrized surface fluxes rather than site-specific measurements. Consequently, the two-film resistance model is applied to predict ammonia and alkylamine emissions from aquatic environments, especially in marine systems.^14,15,29^

However, experimental evaluation of these resistance-based flux estimates for ammonia and amines remains limited, mainly due to the challenges associated with directly measuring these compounds in the gas phase. Their high polarity and reactivity result in substantial sampling losses to instrumentation chamber and inlet surfaces, complicating flux measurements using online, experimental approaches. As a result, previous measurements of amines have often relied on offline collection methods, such as adsorption onto sorbents followed by laboratory analysis.^15,30^ These methods introduce uncertainties related to sampling efficiency, storage, and transport, and are poorly suited for resolving short-term variability in air–water exchange of ammonia and alkylamines.

Consequently, the two-film resistance model has often been applied without direct validation under environmentally relevant conditions, particularly at low wind speeds. Under such conditions (e.g., below ∼ 3.7 m s^–1^),^31^ gas exchange may be dominated by processes not captured by molecular diffusion alone, including convective cooling, surface renewal, and the presence of surfactant layers.^32^ These low-wind regimes are common in freshwater systems, particularly small lakes and rivers, yet remain extremely underrepresented in experimental flux studies.^31,33–35^ Many parametrizations of air–water gas exchange have been developed from marine or large-lake studies conducted at moderate to high wind speeds, typically several m s^–1^.^14,15^ In contrast, small freshwater systems frequently experience much lower effective wind forcing due to limited fetch and sheltering by surrounding vegetation or terrain, with near-calm conditions (<1 m s^–1^) occurring for substantial periods.^31,34^ Targeted laboratory investigations of ammonia and alkylamine fluxes under controlled, low-wind conditions are therefore important for constraining their trace gas exchange in poorly represented low-wind regimes and informing models of air–water exchange in freshwater and other low-wind environments.

In this study, we use a custom water-to-air flux measurement system (FMS) coupled to an atmospheric pressure hydronium chemical ionization mass spectrometer (CIMS) to investigate the exchange behavior of ammonia and alkylamines across the air–water interface of model systems under low-wind conditions (10^–4^ to 10^–3^ m s^–1^). Recent advances in CIMS, particularly using hydronium reagent ions, enable highly sensitive and accurate online detection of ammonia and amines with minimal sampling losses.^36–41^ This system enables continuous, real-time flux measurements from water samples up to 16 L in volume. This study examines fluxes of ammonia (NH_3_), methylamine (MA), dimethylamine (DMA), and trimethylamine (TMA) from model solutions containing their aqueous concentrations of 10^–8^ to 10^–5^ M. These levels are representative of those reported in natural aquatic environments.^19–22^ Using environmentally relevant concentrations enables characterization of realistic emission behavior while isolating the physical and chemical controls on exchange under controlled, low-wind conditions. These observations provide new experimental constraints on the factors governing ammonia and alkylamine transfer across the air–water interface.

## Methods

### Description of the Flux Measurement System

The dimensions and flow operation of the FMS (see Figure 1) are described in Troller et al.,^27^ with key details repeated here. The FMS consists of a 28.5-L perfluoroalkoxy (PFA)-coated stainless-steel tank that can hold up to 16 L of solution. A borosilicate glass sheet with a polytetrafluoroethylene (PTFE) gasket is connected to the top of the tank with ten metal C-clamps. The glass sheet seals the top of the tank, allowing air to flow only through side ports. A controlled and continuous flow rate of humidified zero air (Peak Scientific Precision Zero Air 18L Generator) enters past a PTFE mesh followed by a 16 cm × 5 cm ID stainless-steel tube. The PTFE mesh is used to evenly distribute the incoming flow across the stainless-steel tube. The air then passes through the tank headspace and into a 18 cm × 5 cm ID glass extender connected to the custom-built transverse atmospheric pressure inlet, hydronium ion high resolution CIMS inlet.^42^

**Figure 1:**
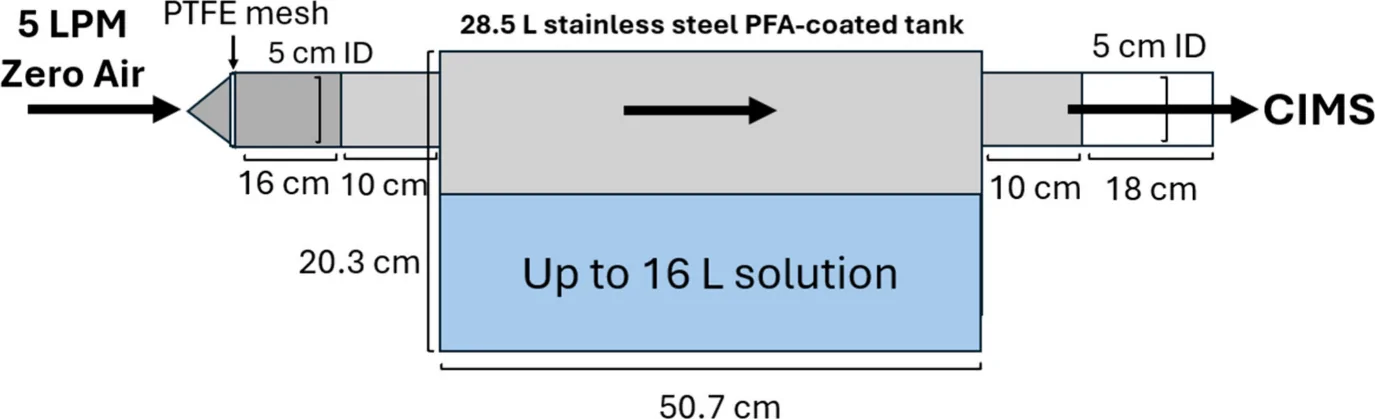
Schematic of water-to-air ammonia and amine flux measurement system (FMS). Total tank volume 28.5 L. Solution volume (blue shade) is typically 16 L.

The FMS was coated in PFA to minimize wall loss of ammonia and alkylamines. Previous studies demonstrate that fluorinated hydrocarbon materials, such as PFA and PTFE, have lower wall loss of polar compounds compared to other materials, due to low reactivity and minimal gas adsorption.^43–45^ Furthermore, glass used in the FMS is predicted to experience considerably lower wall loss in humid conditions compared to dry conditions as the water molecules create a barrier that limits adsorption sites.^43^ Wall loss within the FMS is discussed in further detail below.

The FMS system and CIMS operation settings remained constant throughout the experiments. Although the tank temperature was not actively controlled, it was continuously monitored and did not vary over 1 °C during a single experiment. Overall, the temperature ranged from 19.5 to 25 °C across all flux experiments detailed in this study. These temperature variations, along with background signal behavior, are discussed in greater detail below.

### Measurement of Ammonia and Amines with a Chemical Ionization Mass Spectrometer (CIMS)

The CIMS inlet was connected in-line with the FMS (see Figure 1) to minimize wall losses due to flow constrictions and operated by chemically ionizing a gaseous sample with a specific reagent ion. The ions formed in the atmospheric pressure inlet are then driven into the mass spectrometer via decreasing voltages applied to portions of the inlet. The CIMS operated in positive polarity, using hydronium with water ligands ((H_2_O)_0–3_·H_3_O^+^) as the reagent ions to chemically ionize ammonia and alkylamines. Approximate signal intensities for H_3_O^+^, H_2_O·H_3_O^+^, (H_2_O)_2_·H_3_O^+^, and (H_2_O)_3_·H_3_O^+^ were 3e3, 5e4, 1.5e5, and 5e4 Hz, respectively. Multiple NH_3_ and water ligand peaks (i.e., NH_4_^+^, NH_3_·H_3_O^+^, NH_3_H_2_O·H_3_O^+^, NH_3_(H_2_O)_2_·H_3_O^+^) were observed in the mass spectra, thus NH_3_ concentration and flux are represented as a combination of these signals. MA, DMA, and TMA signals were observed at single peaks at 32.050 amu (CH_3_NH_2_H^+^), 46.065 amu (CH_3_)_2_NH_2_^+^), and 60.080 amu (N­(CH_3_)_3_H^+^), respectively. The ion signal (Hz) for each ionized compound measured in the sample flow is converted to gas concentration by assuming collision rate ionization kinetics with hydronium with water ligands, an assumption that applies well for ammonia and alkylamines.^40,41^ While this specific CIMS configuration was not directly calibrated with ammonia and amine standards for these experiments, previous studies and similar instrument calibrations have shown that ammonia and low-molecular-weight alkylamines ionize near the ion–molecule collision limit in hydronium CIMS systems operated under comparable conditions.^40,41^ The conversion from CIMS ion signal to gas concentration is found in the Supporting Information (SI).

### Background Measurements of the FMS

Prior to any flux experiment, the FMS was triple-cleaned with Alconox detergent, rinsed with excess Elga reverse osmosis purified water (Elga Purelab Flex 2 Water Purification System), and purged with humidified zero air for at least 12 h while measuring background signals. The zero air carrier gas was humidified with high-performance liquid chromatography (HPLC) grade water (Fisher Scientific) to decrease gas wall loss potential, as discussed above. Once background signals were deemed stable (relative standard deviation below 5%), a flux experiment was executed. Instrumental noise was additionally evaluated during stable background periods using the 1σ standard deviation of the ion signals. Noise levels for MA, DMA, and TMA were all below 1 Hz, while ammonia noise was approximately 25 Hz, substantially lower than the observed steady-state background signals (∼10–15 Hz for amines and ∼ 3 × 10^3^ Hz for ammonia), indicating that the measured background signals were well above instrumental noise and represented stable, reproducible signals rather than random fluctuations. Background concentrations from the empty FMS for ammonia, MA, DMA, and TMA averaged 26 pptv, 0.069 pptv, 0.12 pptv, and 0.15 pptv, respectively, with corresponding standard deviations of 17.9 pptv, 0.068 pptv, 0.091 pptv, and 0.18 pptv. Background concentrations varied slightly over time, likely due to residual signals from previous experiments. However, all background signals are accounted for in each experiment, as discussed below. After this background measurement period, either purified water alone or purified water containing added concentrations of ammonia and alkylamines was carefully poured into the tank by opening the glass top. Flux measurements from the solution were then collected for a minimum of 5 h under consistent experimental conditions (e.g., flow rates and instrumental settings).

To ensure the zero air generator was not providing a potential source of ammonia and alkylamines, background signal measurements were also performed using pure nitrogen gas from the headspace of a liquid nitrogen tank. Background ammonia and alkylamine signals in the FMS of pure nitrogen gas were within the same range as observed for zero air (see Figure S1), thus confirming ammonia and alkylamines were not introduced to the experimental system via the zero air carrier gas.

### Ammonia and Alkylamine Fluxes from Elga and Milli-Q Water

Ammonia and alkylamine fluxes were measured from both Elga reverse osmosis purified water and Milli-Q ultrapure Type 1 (Millipore Milli-Q Advantage A10 Water Purification System) water to evaluate background levels of these compounds in purified water sources. While trace ammonia contamination in purified water systems has been previously recognized,^46,47^ quantitative measurements of ammonia and alkylamine backgrounds associated with purified water sources remain limited. Therefore, these experiments were performed to quantify background ammonia and alkylamine levels in purified water and estimate equivalent liquid-phase contamination concentrations, particularly for alkylamines, which have not been widely reported in the literature. These experiments are referred to as Elga blank and Milli-Q blank tests as discussed below. To test whether background ammonia and alkylamine species in Elga reverse osmosis water could be neutralized, 20 μL of sulfuric acid (H_2_SO_4_, 99.999%, Sigma-Aldrich) was added to 16 L of Elga water and the resulting fluxes were compared to those from the Elga blank.

### Variation of Carrier Gas Velocity over the Water’s Surface

Altering gas velocity over the liquid surface was investigated first to provide insights into the system’s proximity to equilibrium in the tank headspace. Aqueous solutions containing ammonia and alkylamines were made from lab-grade solutions of ammonium hydroxide (NH_4_OH, 14.8 M, Reagent ACS, Ward’s Science), methylamine (CH_3_NH_2_, MA, 40% wt. in H_2_O, Sigma-Aldrich), dimethylamine ((CH_3_)_2_NH, DMA, 40% wt. in H_2_O, Acros Organics) and trimethylamine (N­(CH_3_)_3_, TMA, 45% wt. in H_2_O, Sigma-Aldrich) with Elga purified water. Prior to solution preparation, all glassware was cleaned with Alconox detergent and rinsed with Elga water. All target concentrations were prepared by extracting small aliquots of the concentrated stock solutions using a gastight Hamilton syringe and diluting them into a 2 L volumetric flask filled with Elga water. The diluted solution was then transferred into clean 4 L glass jars. This procedure was repeated to prepare a total solution volume of 16 L. The solution containing the dissolved ammonia and alkylamines was first added to the tank, followed by the remaining purified water volumes to promote mixing within the FMS. Ammonia, MA, DMA, and TMA were diluted in 16 L for this experiment to 5.55 × 10^–7^ M, 2.42 × 10^–7^ M, 1.67 × 10^–7^ M, and 1.43 × 10^–7^ M, respectively.

The zero air carrier gas flow rate was varied between 1 and 10 L min^–1^, corresponding to gas velocities 4.8 × 10^–4^ and 4.8 × 10^–3^ m s^–1^, respectively, while keeping total liquid ammonia and alkylamine concentrations and solution volume constant. Gas velocity was calculated assuming uniform flow across the tank cross-section. Thus, reported values represent bulk average velocities, with true surface velocities varying due to flow characteristics and boundary layer development within the chamber. Each flow rate was applied for 25 min before increasing to the next flow rate. In a similar set of experiments, the effect of changing the aqueous solution volume between 8 and 16 L, corresponding to estimated gas velocities of 1.7 × 10^–3^ and 2.4 × 10^–3^ m s^–1^, respectively, while maintaining the zero air carrier gas at 5 L min^–1^ was examined as well.

### Bubbling Zero Air and Nitrogen into the FMS

Bubbling the solution within the FMS with zero air produces aerosol particles which increases the effective water–air interfacial area. As a result, this process is expected to enhance gas flux across the water–air interface.^48,49^ To evaluate this potential effect, flux experiments were performed in which 16 L aqueous Elga water solutions containing 5.5 × 10^–8^ M, 2.4 × 10^–8^ M, 1.6 × 10^–8^ M, and 1.4 × 10^–8^ M of ammonia, MA, DMA, and TMA, respectively, were continuously bubbled with 0.28 L min^–1^ of zero air or pure nitrogen gas. Note that the bubble size distribution was not measured. Although the bubble rise path length was estimated at ∼ 11 cm, bubble characteristics governing interfacial area and residence time were not directly constrained and may affect the resulting fluxes. These experiments were intended to evaluate the overall influence of bubbling on fluxes relative to nonbubbled conditions, rather than to resolve detailed bubble-scale transfer processes.

### Varying Ammonia and Alkylamine Concentrations in the Aqueous Solution

Concentrations ranging from 5.5 × 10^–8^ to 1.9 × 10^–5^ M, 2.4 × 10^–8^ to 1 × 10^–5^ M, 1.6 × 10^–8^ to 8.3 × 10^–6^ M, and 1.4 × 10^–8^ to 8.3 × 10^–6^ M of ammonia, MA, DMA, and TMA, respectively, were added together into 16 L of Milli-Q or Elga purified water. This concentration range captures typical concentrations often found in the natural environment.^19–22^ For these varying liquid concentration flux tests, the zero air flow rate through the FMS was kept constant at 5 L min^–1^. Due to the very low concentration of these solutions, all figures include a 2% uncertainty in total liquid concentration, reflecting the precision limits of the volumetric glassware and syringes used for preparation.

### Ammonia and Amine Water-to-Air Flux Calculation

Ammonia and amine fluxes were calculated using the following equation:

1 $$F_{x},\left(moles,m^{-2},{day}^{-1}\right)=\left(\left[X_{out}\right]-\left[X_{in}\right]\right)\times N_{A}\times \frac{Q}{A}$$

where *F*_*x*_ is the gaseous flux of the analyte, [*X*_*out*_] and [*X*_*in*_] are the outlet and averaged background gas concentrations, respectively, of the analyte measured by the CIMS. The average background concentration, [*X*_*in*_], is defined as the average concentration observed during the 1 h period preceding solution introduction to the FMS. *N*_*A*_ is Avogadro’s number, *Q* is the volumetric flow rate flowing through the FMS headspace, and *A* is the exposed surface area of the water sample in the FMS.

### Two-Film Resistance Model Calculations

Fluxes of ammonia and alkylamines in the FMS were compared to estimated fluxes calculated using the two-film resistance model, which was first applied to the air-ocean interface by Liss and Slater (1974).^29^ The mass transfer of gas-phase species is limited by resistances in both the air and liquid boundary layers. The flux of ammonia and amines, *F* (moles m^–2^ day^–1^), is calculated using the following main equation:

2 $$F,\left(moles,m^{-2},{day}^{-1}\right)=k_{t}\left(C_{w}-C_{g}/H\right)$$

where *k*_*t*_ is the overall air–water gas transfer velocity (m day^–1^), *C*_*w*_ is the dissolved concentration of the compound in water (mol m^–3^), *C*_*g*_ is the gas-phase concentration in air (mol m^–3^), and *H* is the dimensionless Henry’s law constant describing the equilibrium partitioning between the aqueous and gas phases. Further details of each parameter can be found in the SI.

### Henry’s Law Gaseous Concentration

Gaseous concentrations reported in this work are also compared to concentrations estimated by Henry’s Law. Henry’s Law states that at a constant temperature, the amount of gas that dissolves in a liquid is directly proportional to the partial pressure of that gas above the liquid at equilibrium. This relationship is represented mathematically as

3 $$C_{w}=H_{s}^{cp}P,\left(mol,L^{-1}\right)$$

where *C*_*w*_ is the liquid concentration of the dissolved, unprotonated gas, *H*_*s*_^*cp*^ (mol L^–1^ atm^–1^) is the Henry’s Law solubility constant specific to the gas–liquid pair, and *P* (atm) is the partial pressure. *H*_*s*_^*cp*^ values were previously determined via experiments or theory and are represented as a range in this work (see Table S1).^50^

Henry’s Law assumes the gas is at low concentration, does not chemically react with the solvent, and both the gas and solvent behave ideally. Henry’s Law solubility constants, *H*_*s*_^*cp*^, are also temperature dependent. The FMS tank temperature ranged from 19.5 to 25 °C across all experiments, while temperature variation during any individual experiment was less than 1 °C. Temperature is known to influence ammonia and alkylamine solubility and therefore affects Henry’s Law partitioning behavior. Across the full experimental temperature range, the corresponding variation in Henry’s Law solubility constants is estimated to be approximately 30%. Given the substantial variability (i.e., over an order of magnitude) in published Henry’s Law constants reported in the literature (Table S1), this temperature dependence is assumed to be largely encompassed within the broader Henry’s Law ranges represented in this work.

Additionally, the liquid concentration of the dissolved, unprotonated ammonia and alkylamines is highly dependent on the pH of the water. The following Henderson–Hasselbalch equation is used to calculate the unprotonated concentration of ammonia and alkylamines:

4 $$C_{w}=C_{tot}\times 10^{\left({pH}_{w}-pKa\right)/(1+10^{\left({pH}_{w}-pKa\right)})}$$

where *C*_*tot*_ is the total dissolved concentration, accounting for both protonated and unprotonated forms, *pH*_*w*_ is the pH of the aqueous solution, and *pK*_*a*_ is the acid-dissociation constant (see Table S1). The pH of aqueous solutions used in flux experiments ranged from 6 to 6.7, measured using a Hanna edge Dedicated pH/ORP meter. Thus, due to the variability in Henry’s Law solubility constants, water pH, and experimental temperature, Henry’s Law theoretical gaseous concentrations are represented as a range in this work. Effects of salinity on *C*_*w*_ are not accounted for due to all water samples having a salinity of 0 ppt. The present work intentionally focused on simplified low-wind experiments using purified water to isolate and constrain the complex parameters influencing trace gas exchange behavior under near-stagnant conditions. However, in high salinity environments, such as ocean systems, increased ionic strength will reduce the solubility of ammonia and alkylamines via salting-out effects. These saline conditions can also shift acid–base equilibria, potentially enhancing the gas transfer from water to air.^51^ Future experiments should therefore examine broader environmentally relevant conditions, including varying pH, salinity, ionic composition, and more complex aqueous matrices representative of natural water bodies.

## Results and Discussion

### Single Flux Experiment Gas Concentration and Flux Time Series

Figure 2 shows the gaseous concentration and flux time series for a single experiment performed on a 16 L aqueous solution consisting of ammonia, TMA, DMA, and MA. Stable background gas concentrations of ammonia, TMA, DMA, and MA are shown before time 0 h. Gaseous concentrations of these compounds initially spiked and increased 1 to 2 orders of magnitude above background concentrations when the aqueous solution was poured into the FMS at time 0 h. The rapid increase in gaseous ammonia and amine concentrations, reaching peak concentrations within approximately 5 min of solution introduction, suggests that wall adsorption within the FMS does not dominate the initial gas-phase response under these experimental conditions. Most experiments were conducted for approximately 5 h, while a subset of experiments were extended to 24 h to evaluate longer-term variability and concentration stability within the system. During all experiments, gaseous ammonia and alkylamine concentrations gradually decreased and, in some cases, approached a plateau. Since the reagent signals remained constant, the decrease in gas concentration is not due to reagent signal decay. This gradual decrease more likely reflects a combination of declining dissolved concentrations resulting from continued water-to-air transfer and adsorption/desorption interactions with the PFA-coated FMS surfaces over longer experimental time scales. The observed behavior, consisting of an initial decrease followed by the development of a plateau in some experiments, suggests that wall interactions may be most important early in the experiment and diminish with time. Such behavior is consistent with a finite-capacity sink in which adsorption sites on the PFA-coated tank surfaces become progressively occupied with compounds, reducing the net wall-loss rate and allowing gas-phase concentrations to approach a quasi-steady state. Although the specific species responsible for conditioning these surfaces cannot be determined from the present experiments, adsorption of ammonia, amines, water, or other trace contaminants may contribute to this behavior.

**Figure 2:**
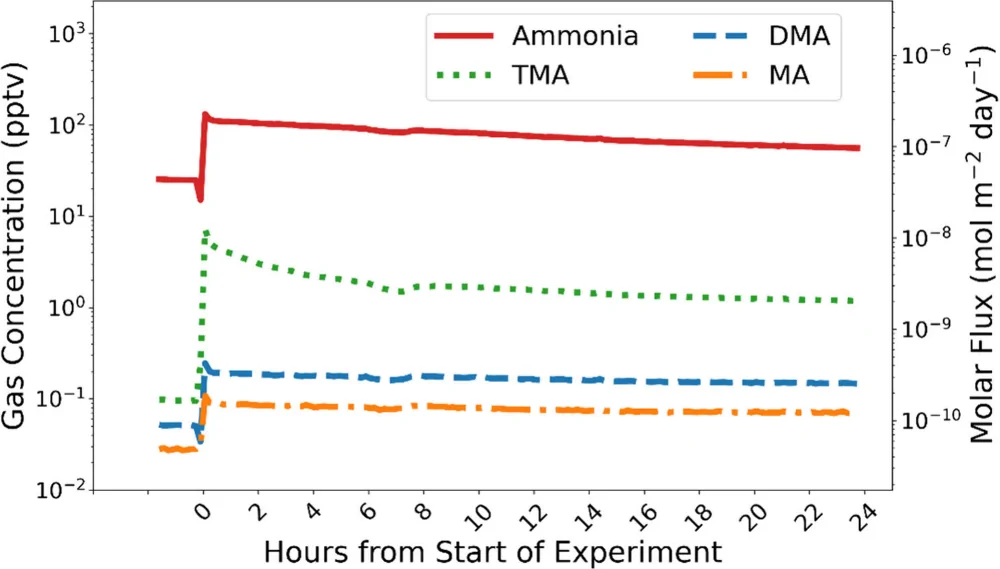
10 min averaged gaseous concentrations of compounds during one flux experiment. Total liquid concentrations of ammonia, TMA, DMA, and MA are 4.6 × 10^–6^ M, 9.5 × 10^–7^ M, 8.3 × 10^–7^ M, and 8.1 × 10^–7^ M, respectively.

Changes in solution pH could also influence gas-phase concentrations by altering the fraction of dissolved ammonia and amines present in their unprotonated forms. As volatilization removes dissolved ammonia and amines, the acid–base equilibria shift toward the neutral species, which tends to increase solution pH and partially offset the loss of volatile compounds. Solution pH was measured prior to each experiment and ranged from approximately 6.0 to 6.7 across all experiments, with measured values incorporated directly into the Henderson–Hasselbalch calculations used for the Henry’s Law predictions discussed throughout the manuscript. As the experiments were conducted using dilute ammonia and alkylamine concentrations in purified water and only a small fraction of the dissolved compounds volatilized during a typical 5 h experiment, substantial pH drift is not expected. Furthermore, pH-driven changes would be expected to produce a more gradual and continuous evolution in gas-phase concentrations rather than the observed tendency toward a plateau, suggesting that surface interactions likely play a more important role in the long term concentration behavior.

TMA typically exhibited a sharp initial spike in gas concentration, increasing nearly 2 orders of magnitude above background levels, shortly after the solution was introduced to the FMS, followed by a steady decline of over half a magnitude over the 24 h flux measurement period. In comparison, MA and DMA typically showed smaller initial increases and reached near-constant concentrations within the first few hours, with only minor steady decreases observed over the remainder of the experiment. Ammonia followed a similar trend to MA and DMA, with a rapid rise and a more gradual decline toward steady values. These differences in temporal behavior can be attributed to variations in volatility and surface activity among the alkylamines. TMA has both a high vapor pressure and high surface activity compared to MA and DMA, allowing for more rapid transfer into the gas phase while also being enriched at the air–water interface.^8,52^ MA and DMA, in contrast, are less volatile and have slightly lower surface activity due to lower hydrophobicity, leading to slower and more stable fluxes over time. Ammonia continuously had higher background signals (10^0^ to 10^1^ pptv) compared to the alkylamines, likely due to ammonia being more abundant in the zero air carrier gas and room air when the tank was opened for 1 min to pour in aqueous solutions.

### Varying Zero Air Carrier Gas Velocity over Liquid Surface

To evaluate if the two-film resistance model applies well under low wind conditions, we conducted controlled flux experiments in our FMS system that altered the wind velocity over the solution’s surface. Figure 3 displays results from duplicate experiments that observe the gas concentration and molar flux response of ammonia and the alkylamines while changing the flow rate of the zero air carrier gas through the FMS headspace above an Elga water solution containing 5.55 × 10^–7^ M, 2.42 × 10^–7^ M, 1.67 × 10^–7^ M, and 1.43 × 10^–7^ M of ammonia, MA, DMA, and TMA, respectively. The average gas concentration and flux over the 25 min intervals are shown in Figures 3a and 3b, respectively. Increasing the zero air carrier gas flow rate from 1 to 10 L min^–1^, corresponding to surface velocities of 4.8 × 10^–4^ and 4.8 × 10^–3^ m s^–1^, consistently led to a near constant observed gas concentration. However, the calculated flux increases because the CIMS flux eq (eq 1) incorporates the flow rate as a dilution term. Since the measured concentration remains constant as dilution increases, the emitted flux must increase accordingly. This behavior indicates enhanced flux emission rather than attainment of equilibrium under low-flow conditions.

**Figure 3:**
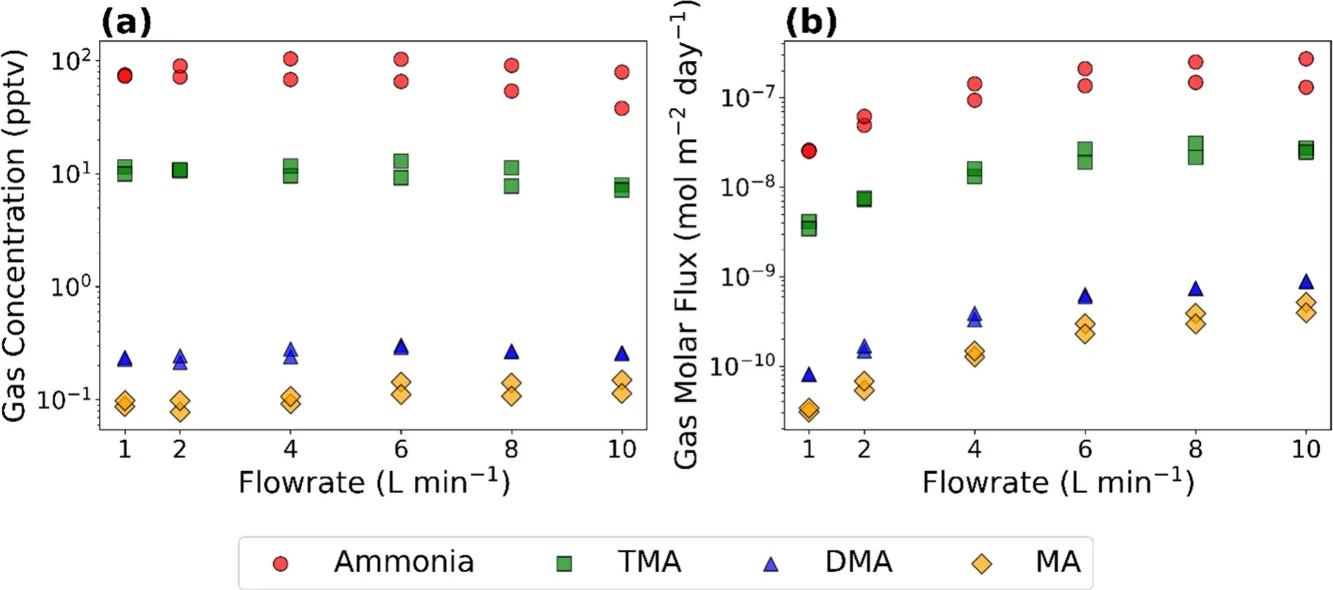
(a) Gas concentrations and (b) molar flux of ammonia (red circles), TMA (green squares), DMA (blue triangles), and MA (yellow diamonds) while varying zero air carrier gas flow rate over 16 L Elga water solutions.

Given the near-stagnant flow conditions in the FMS tank, the two-film resistance model is not appropriate and fails to reproduce the observed CIMS fluxes for all compounds examined. Specifically, when the model is applied using the unprotonated aqueous concentrations and corresponding gas-phase concentrations measured by the CIMS (see SI for two-film resistance model calculations), it typically predicts fluxes of the opposite direction or overestimates the fluxes by orders of magnitude (see Figure S2). This indicates that the model is not appropriate for the near-stagnant, low-wind conditions of the FMS.

According to Henry’s Law, the gas–liquid interface should reflect equilibrium conditions, with no net flux when equilibrium is achieved. However, because the FMS continuously introduces and removes carrier gas from the tank headspace, slight deviations from ideal equilibrium conditions are expected. The observed positive flux values at all flow rates therefore indicate a persistent net transfer of these compounds from liquid to gas despite the near-stagnant conditions within the FMS. Nevertheless, our data suggests that gas exchange in the FMS more closely follows equilibrium-driven behavior, where the driving force for flux is primarily governed by partitioning by Henry’s Law rather than boundary layer resistance. This supports the use of Henry’s Law as a more appropriate method for interpreting fluxes in low-wind systems like in the FMS.

Additionally, as the volumetric flow rate increases up to 10 L min^–1^, the current FMS geometry likely introduces increasingly complex headspace fluid dynamics. Higher inlet flow rates may result in preferential jetting of air across portions of the tank headspace and liquid surface while leaving other regions comparatively stagnant. This behavior could reduce the effective residence time of air over portions of the liquid surface and limit the uniform partitioning of emitted ammonia and amines into the sampled headspace. As a result, the system likely does not exhibit the increasingly exponential gas exchange response theoretically expected at higher flow-driven exchange rates, as portions of the carrier gas may bypass effective equilibration with the liquid surface. Thus, the observed plateauing behavior at higher flow rates likely reflects limitations of the current experimental configuration and tank flow geometry rather than purely equilibrium-controlled exchange behavior.

In addition to varying the zero air carrier gas flow rate, solution volume variations were tested to similarly alter the wind velocity over the solution’s surface. Such flux experiments were performed with 8 and 16 L Elga water solutions containing the aqueous concentrations of 5.5 × 10^–8^ M, 2.4 × 10^–8^ M, 1.6 × 10^–8^ M, and 1.4 × 10^–8^ M of ammonia, MA, DMA, and TMA, respectively, under identical experimental and operational conditions. Increasing the volume of the aqueous solution decreases the available tank headspace, increasing the effective wind speed due to the reduced cross-sectional area within the FMS. However, there was a negligible difference in measured gas concentration observed with the 8 and 16 L aqueous solutions, corresponding to gas velocities of 1.7 × 10^–3^ to 2.4 × 10^–3^ m s^–1^, respectively (see Figure S3). Notably, the 8 L aqueous volume increases the exposed internal surface area of the FMS relative to the 16 L aqueous volume. The absence of a significant difference in gas-phase concentrations therefore indicates that wall losses within the FMS are also minimal under these conditions. Moreover, across the broader low-wind regime examined in Figure 3, where carrier gas flow rates were varied from 1 to 10 L min^–1^, the corresponding order-of-magnitude increase in estimated wind speed produced no measurable change in gas-phase concentrations, indicating that flux rates are not strongly controlled by wind and supporting near-equilibrium between the aqueous and gas phases.

### Negligible Gas-Phase Concentration Difference when Bubbling Aqueous Solution

To assess whether water-to-air exchange in the FMS was limited by interfacial transport, the effect of bubbling on measured gas-phase concentrations was evaluated. A 16 L aqueous solution containing 5.5 × 10^–8^ M, 2.4 × 10^–8^ M, 1.6 × 10^–8^ M, and 1.4 × 10^–8^ M of ammonia, MA, DMA, and TMA, respectively, was bubbled from the bottom of the tank with 280 cm^3^ min^–1^ of dry zero air or dry N_2_. Bubbling introduces additional complexities by creating aerosols and increasing the effective gas–liquid interfacial surface area. Bubble-induced turbulence also enhances surface renewal and generates more complex fluid dynamics at the liquid surface.^48,49^ As a result, these experiments were interpreted using measured gas-phase concentrations rather than converted fluxes. Despite this additional mixing, no significant change in the gas-phase concentrations in the FMS headspace above the aqueous solution of any compound was observed compared to nonbubbling experiments (Figure S4).

Gas-phase concentrations of NH_3_, MA, DMA, and TMA measured during bubbling experiments differed by no more than ∼ 35% (either higher or lower depending on the compound) from nonbubbling experiments when averaged over the 2 h experiment (Figure S4). These differences fall within the typical uncertainty associated with CIMS-derived concentration measurements, which is commonly taken to be a factor of 2.^40^ The ranges of measured concentrations during the three conditions overlapped substantially throughout the experiments. For ammonia, average concentrations varied by no more than 20–30 pptv relative to nonbubbling conditions, while alkylamine average concentrations differed by ≤ 0.02 pptv, ≤ 0.05 pptv, and ≤ 2 pptv for MA, DMA, and TMA, respectively. These small and overlapping differences indicate that additional interfacial mixing and surface area generated by bubbling did not measurably enhance volatilization under the conditions examined. Thus, the observed fluxes were not constrained by gas-phase transport resistance but instead reflect near-equilibrium.

### Varying Ammonia and Alkylamine Liquid Concentrations in Model Aqueous Solutions

Figures 4–7 present gas-phase concentrations measured by the CIMS in the FMS headspace above aqueous solutions containing combined ammonia, MA, DMA, and TMA across a range of liquid-phase concentrations. These figures also include gas-phase concentrations measured above purified water blanks (i.e., Elga and Milli-Q water) and Elga water containing 20 μL sulfuric acid, corresponding to 2.3 × 10^–5^ M sulfuric acid. Gas concentrations are reported as 5 h averages following the solution introduction into the FMS. Under equilibrium conditions, Henry’s Law predicts that increasing aqueous concentrations should result in proportional increases in gas-phase concentrations for each compound. Accordingly, these measurements provide a direct test of the extent to which gas–liquid partitioning in the FMS follows Henry’s Law behavior and whether deviations from this expectation occur under the following experimental conditions.

**Figure 4:**
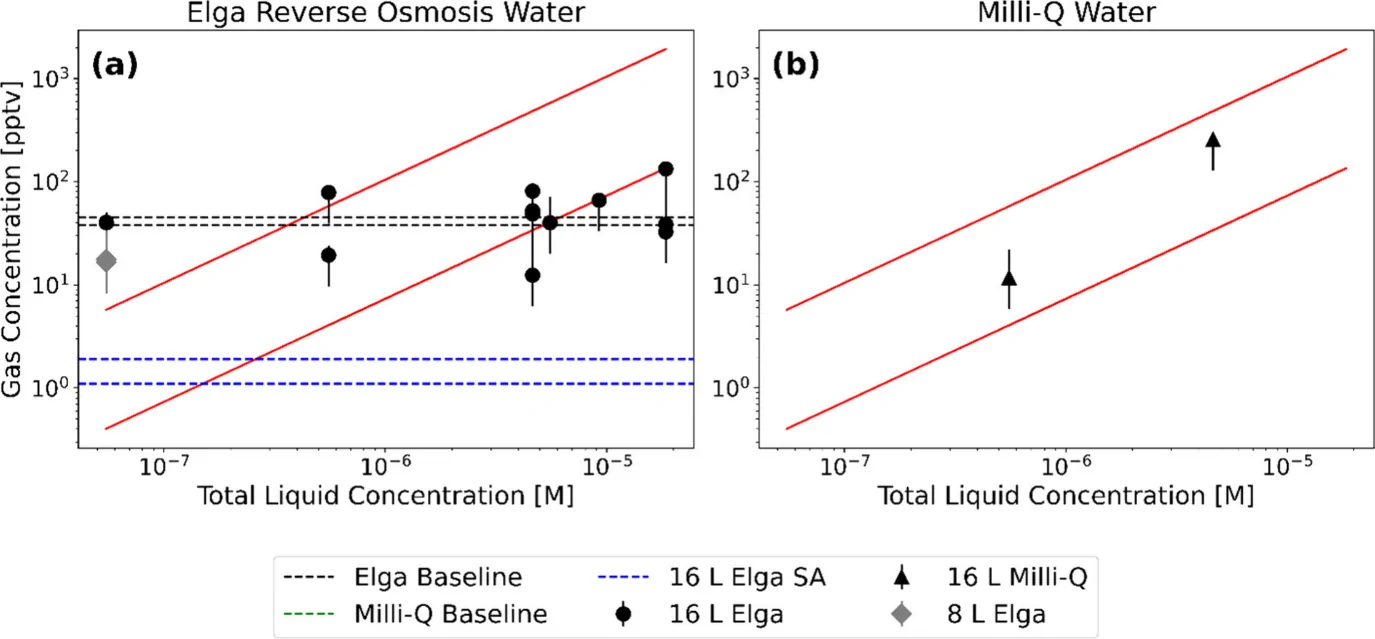
Ammonia exchange from aqueous solutions. The points show CIMS-based concentrations of zero air passed over the surface of varied concentrations of ammonia in solution in (a) Elga reverse osmosis water, and (b) Milli-Q ultrapure water. The points show 5 h average gas concentrations, with associated concentration ranges measured throughout the 5 h experiments. Different volumes of water were added to the FMS tank: 8 L Elga water (gray diamonds), 16 L Elga water (black dots), and 16 L Milli-Q water (black triangles). Dashed lines represent the gas concentrations measured from 16 L Elga water blank (black), and 16 L Elga water with 20 μL sulfuric acid (SA, blue). Note for the Milli-Q water blank, measured concentrations fell below the background signal measured in the empty FMS and thus are not shown in the figure. Predicted concentrations based on the range of Henry’s Law values for ammonia are represented in the region between the red lines.

**Figure 5:**
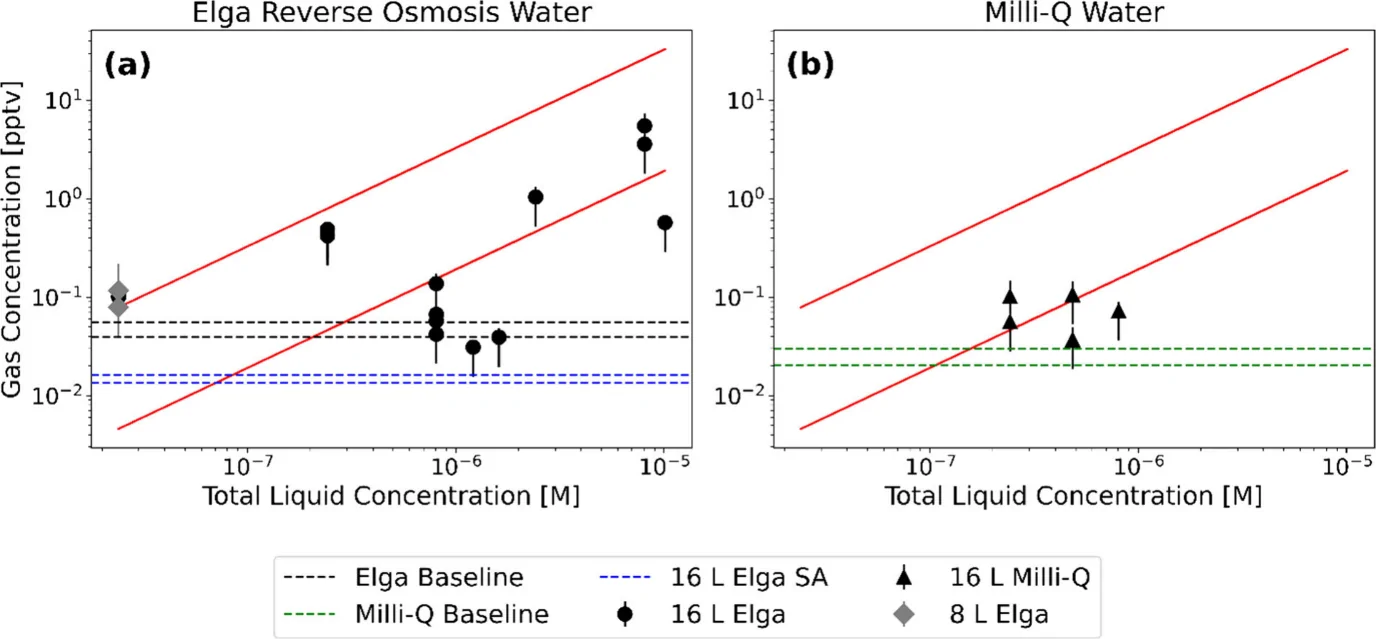
Methylamine exchange from aqueous solutions. Comparison of CIMS experimental 5-h average gas concentrations (symbols) from (a) Elga reverse osmosis water and (b) Milli-Q ultrapure water for MA. Henry’s Law theoretical gas concentrations are represented as an estimated range (red lines). 8 L Elga tests (gray diamonds), 16 L Elga water tests (black dots), and 16 L Milli-Q water tests (black triangles) are associated with varying liquid concentration. Dashed lines represent the gas concentrations measured from 16 L Elga water blank (black), 16 L Elga water with 20 uL SA (blue), and 16 L Milli-Q water blank (green).

**Figure 6:**
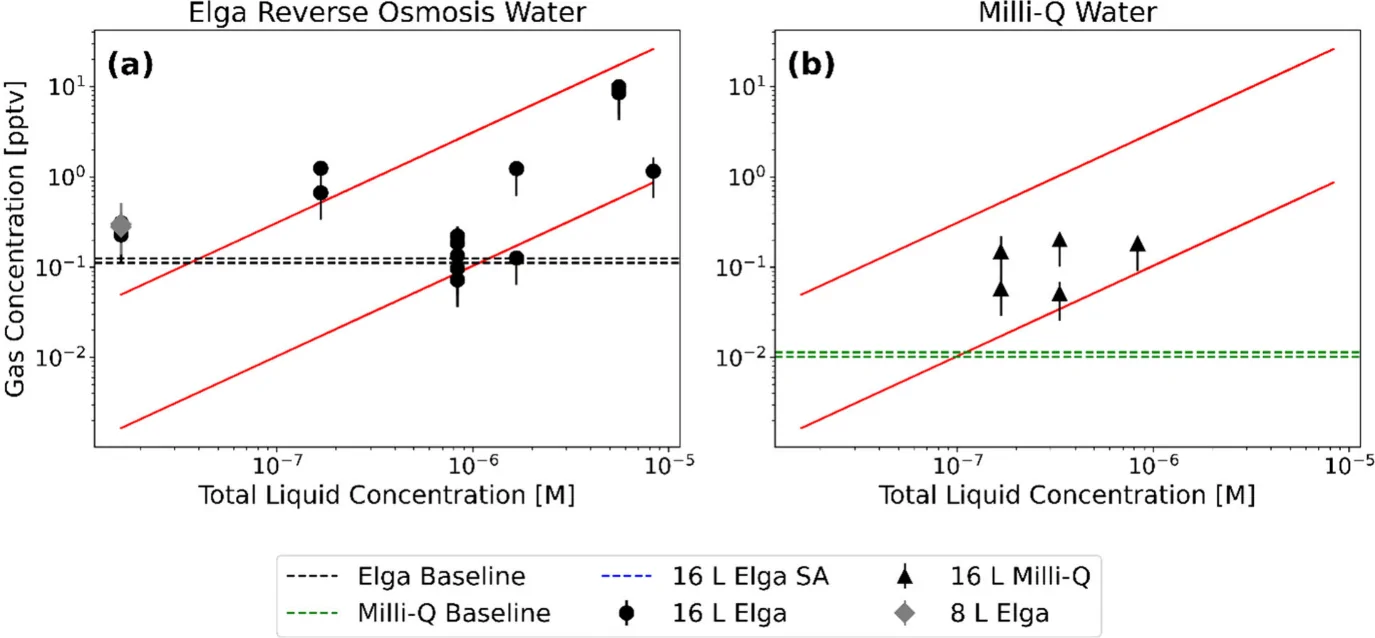
Dimethylamine exchange from aqueous solutions. Comparison of CIMS experimental 5 h average gas concentrations (symbols) from (a) Elga reverse osmosis water and (b) Milli-Q ultrapure water for DMA. Henry’s Law theoretical gas concentrations are represented as an estimated range (red lines). 8 L Elga tests (gray diamonds), 16 L Elga water tests (black dots), and 16 L Milli-Q water tests (black triangles) are associated with varying liquid concentration. Dashed lines represent the gas concentrations measured from 16 L Elga water blank (black) and 16 L Milli-Q water blank (green).

**Figure 7:**
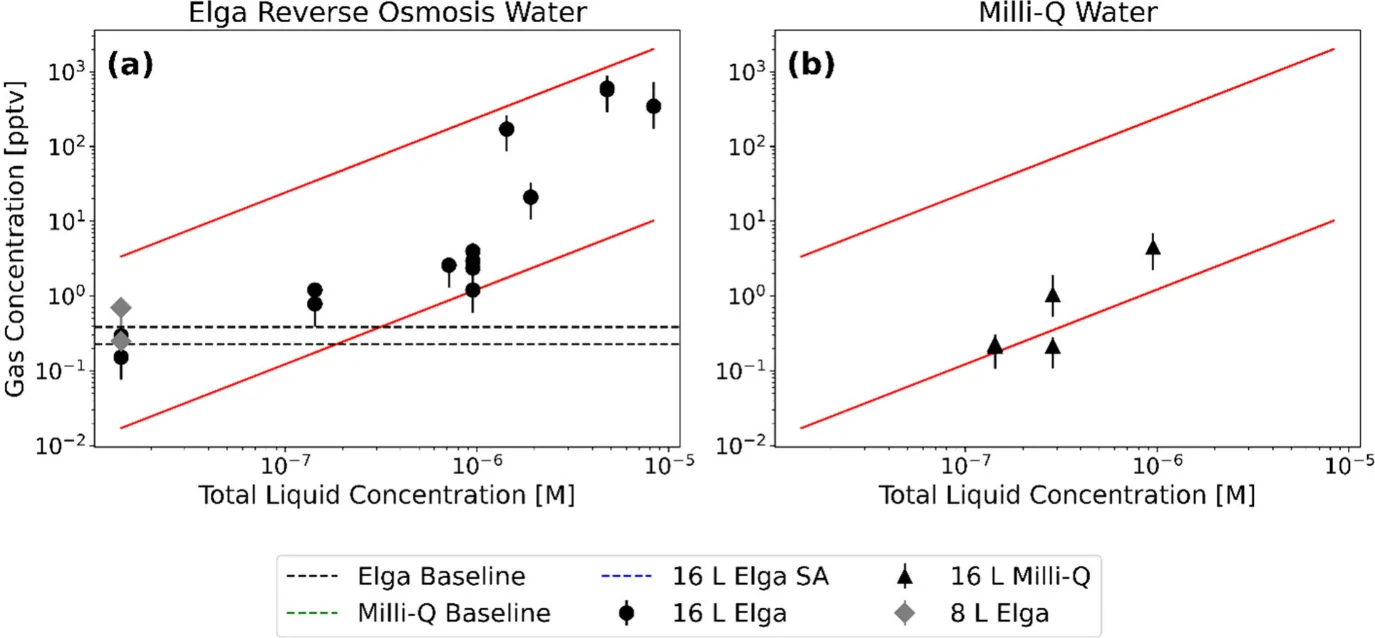
Trimethylamine exchange from aqueous solutions. Comparison of CIMS experimental 5 h average gas concentrations (symbols) from (a) Elga reverse osmosis water and (b) Milli-Q ultrapure water for TMA. Henry’s Law theoretical gas concentrations are represented as an estimated range (red lines). 8 L Elga tests (gray diamonds), 16 L Elga water tests (black dots), and 16 L Milli-Q water tests (black triangles) are associated with varying liquid concentration. Dashed lines represent the gas concentrations measured from 16 L Elga water blank (black).

### Ammonia

Figure 4 displays the relationship between total liquid ammonia concentrations and corresponding gas concentrations measured in the FMS headspace, with the Henry’s Law predicted range. Experiments were conducted using Elga and Milli-Q water, shown in panels (a) and (b), respectively. In Elga blank experiments (Figure 4a) with no added ammonia, headspace concentrations averaged about 41 pptv after background subtraction, which refers to the subtraction of the clean, empty FMS background signals measured prior to solution introduction. A gas concentration of 41 pptv ammonia corresponds to a Henry’s Law predicted aqueous concentration range of 3.9 × 10^–7^ to 5.6 × 10^–6^ M, indicating that Elga water contains background ammonia within this range. The Milli-Q blank experiments (Figure 4b) resulted in gas concentrations lower than the clean-tank background, implying that Milli-Q water acted as a sink for the trace ammonia inside the FMS. When 20 μL of sulfuric acid was added to 16 L of Elga water, the gas-phase ammonia concentration dropped dramatically to an average of 1.5 pptv above background levels. This trend is consistent with acid–base equilibria shifting ammonia into ammonium and confirms that the system is sensitive to small changes in solution chemistry, such as shifts in pH.

In experiments where dilute ammonia (5.5 × 10^–8^ to 1.9 × 10^–5^ M) was added to each purified water type, the measured headspace concentrations showed little correlation with total liquid concentration. While Elga water experiments consistently produced measurable signals above empty tank background signals, they did not scale with aqueous concentration. For Elga water (Figure 4a), gas-phase ammonia ranged between 16 to 250 pptv with little influence from increasing aqueous ammonia concentrations. This demonstrates that small increases in aqueous ammonia concentration did not overcome the variable contributions from the Elga water alone. Conversely, Milli-Q water experiments often yielded no detectable enhancement above empty FMS signals. Milli-Q experiments (Figure 4b) displayed even greater variability where only two of five experiments performed with 16 L solutions containing up to 5 × 10 M aqueous ammonia produced ammonia gas concentrations above the empty FMS signals. The remaining trials fell below the empty tank background concentrations. These results highlight that the high background ammonia within the FMS, combined with the distinct behaviors of different ultrapure waters, complicates the interpretation of gas–liquid partitioning at low ammonia concentration levels.

Similar to the effects observed in ultrapure water, ammonia is known to be pervasive in indoor air environments, with indoor air concentrations observed in past studies ranging from 10 to 70 parts per billion by volume (ppbv).^53–55^ Using the range of Henry’s Law solubility constants applied in this work, these ambient room air values correspond to an aqueous-phase equilibrium concentration of approximately 1 × 10^–4^ to 1 × 10^–2^ M, exceeding the concentrations of ammonia added to the water in these experiments by more than an order of magnitude. This helps explain why adding up to 10^–5^ M ammonia to Elga and Milli-Q waters had a minimal effect on the observed gas-phase concentration. Thus, these experiments demonstrate that even ultrapure water sources contain or quickly equilibrate with enough ammonia in the air that additions of up to 10^–5^ M have little effect on the gas-phase concentration. Only significant chemical changes, such as pH adjustment, meaningfully shift ammonia gas-phase concentrations in this controlled system.

### Methylamine

Figure 5 presents measured gas-phase MA concentrations as a function of total liquid concentrations for Elga and Milli-Q water, along with Henry’s Law predicted values. Blank water tests showed that both Elga (Figure 5a) and Milli-Q (Figure 5b) water sources contained measurable MA above the FMS background signal. In Elga blank experiments, gas-phase MA concentrations remained low averaging at 0.05 pptv, corresponding to a Henry’s Law predicted aqueous concentration range of 1.5 × 10^–8^ to 2.3 × 10^–7^ M. Milli-Q blanks yielded slightly lower signals averaging at 0.03 pptv, corresponding to a Henry’s Law predicted aqueous concentration range of 9.2 × 10^–9^ to 1.6 × 10^–7^ M. This indicates that background MA in both water sources contributes to the headspace concentrations. The addition of 20 μL of sulfuric acid to the 16 L Elga water (i.e., 2.3 × 10^–5^ M SA) reduced MA gas concentrations relative to the Elga blank to 0.015 pptv, consistent with neutralization of dissolved MA and further supporting the presence of background MA in the water.

When dilute MA was added to each water source, gas-phase concentrations generally increased with total liquid concentration but showed notable thresholds and variability. For Elga water (Figure 5a), gas-phase MA remained within 0.45 pptv of the Elga blank until liquid concentrations exceeded approximately 6 × 10^–6^ M, suggesting this may be the threshold at which added aqueous MA begins to quantifiably influence gas-phase levels above background MA from Elga water alone. Milli-Q experiments, conducted over a narrower concentration range (Figure 5b), produced gas-phase concentrations similar to Elga water at comparable concentrations, though slightly elevated relative to Milli-Q blanks.

Overall, measured MA gas concentrations often fell slightly below Henry’s Law predictions, particularly at lower concentrations, and exhibited scatter likely due to random errors. These errors include minor differences in dilution or variations in background MA in the water sources over the course of experiments spanning nearly two years. Additionally, because each solution was exposed to room air while dispensing from the water purification systems and pouring into the tank, variations in ambient room air MA concentrations could shift the resulting headspace measurements, adding to the scatter observed in the MA gas-phase concentrations. Though ambient air MA concentrations were not directly measured in this study, past studies have measured pptv to tens of pptv levels of individual amines in ambient air.^8,36,38^ Assuming a room-air MA concentration of 1–10 pptv, the corresponding Henry’s Law equilibrium yields an aqueous MA concentration of approximately 5 × 10^–6^ M to 5 × 10^–5^ M, respectively, if equilibrium were reached. Aqueous MA concentrations could still increase without reaching equilibrium but would be expected to be below these equilibrium values. Similar to ammonia, this overlaps and exceeds the amount of MA added to the water sources. As a result, MA displayed the greatest measurement variability among the amines studied, underscoring the importance of precisely characterizing the concentration of aqueous MA when evaluating its water-to-air flux.

### Dimethylamine

Figure 6 shows measured gas-phase concentrations of DMA as a function of total aqueous concentration, alongside the predicted range from Henry’s Law. Blank water tests indicated that both Elga and Milli-Q waters contained measurable DMA above the empty FMS background. The Elga blank (Figure 6a) exhibited higher gas-phase dimethylamine (DMA) concentrations, with an average of 0.12 pptv, compared to the Milli-Q blank (Figure 6b), which averaged 0.01 pptv. Based on Henry’s Law, these gas-phase concentrations correspond to predicted aqueous DMA concentration ranges of 3.9 × 10^–8^ to 1.2 × 10^–6^ M for Elga water and 3.2 × 10^–9^ to 9.7 × 10^–8^ M for Milli-Q water. These results indicate the presence of measurable background DMA in both water types, while confirming the higher purity of Milli-Q water relative to Elga water. When 20 μL of sulfuric acid was added to Elga water, DMA gas concentrations fell below empty FMS background levels in duplicate tests, consistent with neutralization of aqueous DMA and demonstrating that the system is sensitive to changes in solution chemistry.

Subsequent experiments with varying DMA liquid concentrations (10^–9^ to 10^–5^ M) showed that 14 out of 17 Elga water tests produced gas-phase DMA above the Elga blank, while all Milli-Q tests yielded concentrations above the Milli-Q blank. Gas-phase DMA largely fell within or near the Henry’s Law predicted range, though some tests did not show a clear positive correlation with liquid concentration. For example, several experiments at approximately 8 × 10^–7^ M consistently produced gas concentrations around 10^–1^ pptv, while other experiments at approximately 2 × 10^–7^ M produced concentrations closer to 1 pptv. In contrast, at aqueous DMA concentrations above ∼ 10^–6^ M, a clearer positive relationship between liquid-phase and gas-phase concentrations was observed. The variability at lower aqueous concentrations likely reflects sensitivity to fluctuating background DMA present in the water. Additionally, despite the narrower concentration range for Milli-Q tests, gas-phase results were generally comparable to those from Elga water at similar concentrations, indicating that both water types behave similarly when trace DMA is added.

### Trimethylamine

Figure 7 displays gas-phase TMA concentrations as a function of total aqueous concentration, alongside the Henry’s Law predictions. Blank water tests showed clear differences between the two ultrapure water sources. In Elga blank experiments (Figure 7a), measurable TMA was present in the headspace, with an average concentration of 0.3 pptv, which corresponds to a Henry’s Law predicted aqueous concentration range of 1.3 × 10^–9^ to 2.5 × 10^–7^ M. In contrast, Milli-Q blank tests (Figure 7b) produced gas-phase concentrations below the background signal and therefore are not shown in Figure 7b, suggesting that Milli-Q water may act as a sink for background TMA. When 20 μL of sulfuric acid was added to Elga water, TMA gas concentrations dropped below the background level and are therefore not shown in Figure 7a, indicating effective neutralization of aqueous TMA and possible uptake of background gaseous TMA by the aqueous phase.

When varying TMA liquid concentrations were added to the water sources (10^–8^ to 10^–6^ M), both Elga and Milli-Q tests showed positive correlation between aqueous-phase and gas-phase TMA. For Elga water, all liquid concentrations ≥ 10^–8^ M produced gas-phase TMA above the Elga blank. A power-law regression between aqueous-phase and gas-phase TMA concentrations in Elga water yielded an R^2^ value of 0.731, indicating a strong positive relationship between liquid and gas-phase TMA under the experimental conditions examined. The 1.4 × 10^–8^ M test yielded a gas-phase concentration comparable to the blank, implying that at very low concentrations, the added TMA does not measurably influence the background TMA already present in the system.

Among all compounds examined, TMA exhibited the best agreement with Henry’s Law predictions in Elga water, remaining within the predicted range and showing a clear concentration-dependent response. Additionally, unlike ammonia, MA, and DMA, measured gas-phase TMA concentrations were minimally influenced by room-air background. This behavior likely results from TMA’s higher surface activity and greater hydrophobicity compared to the smaller amines, which enhance its accumulation at the air–water interface and promote volatilization.^56^ In contrast, stronger hydration and bulk retention of MA and DMA may impede effective gas–liquid equilibration at low concentrations, whereas the weaker hydration of TMA likely enables more reproducible equilibrium behavior and closer agreement with Henry’s Law predictions. Milli-Q tests, conducted over a narrower range of aqueous concentrations, likewise showed a positive trend between liquid and gas-phase TMA. Combined, these results highlight the strong air–water exchange behavior of TMA and underscore the importance of accurately determining aqueous concentrations when interpreting its gas-phase measurements. Lastly, using the measured gas-phase concentrations and calculated unprotonated aqueous TMA concentrations, apparent experimental Henry’s Law constants were estimated to range from approximately 0.7 to 1.5 × 10^3^ M atm^–1^, with mean and median values of ∼ 2.8 × 10^2^ and ∼ 75 M atm^–1^, respectively. This range is far broader than reported in previous literature (Table S1). However, the FMS was continuously flushed with zero air carrier gas and may not have achieved true thermodynamic equilibrium. Therefore, these values should be interpreted cautiously as apparent experimental partitioning constants rather than definitive equilibrium Henry’s Law constants. Additional experiments under fully closed or equilibrium-controlled conditions would be valuable for further constraining literature Henry’s Law values for TMA and the other compounds examined in this study.

## Conclusions

This study provides direct experimental constraints on the water-to-air exchange behavior of ammonia and alkylamines under low-wind conditions using a custom flux measurement system coupled to online hydronium CIMS. Across all experiments, measured gas-phase concentrations and fluxes deviated systematically from predictions based on the two-film resistance model, demonstrating that this framework is not appropriate for near-stagnant conditions representative of many freshwater environments. Instead, observed emissions were largely governed by near-equilibrium partitioning, with Henry’s Law providing a more appropriate basis for interpreting gas–liquid exchange under these conditions.

Our results further show that background contamination in commonly used purified water sources exerts a strong influence on measured gas-phase concentrations at environmentally relevant levels. Both Elga reverse osmosis and Milli-Q ultrapure water contained measurable background ammonia and alkylamines, with consistently lower concentrations observed in Milli-Q water. For ammonia and the smaller alkylamines (i.e., MA and DMA), background contributions and equilibration with ambient laboratory air often dominated the gas-phase signal, obscuring the effects of minute aqueous additions. In contrast, TMA exhibited clearer concentration-dependent behavior and closer agreement with Henry’s Law predictions, consistent with its weaker hydration and greater surface activity.

Together, these findings highlight the importance of directly characterizing background levels, solution chemistry, and equilibrium behavior when measuring and modeling ammonia and alkylamine emissions from aquatic systems. The results demonstrate that commonly applied resistance-based models can substantially misrepresent fluxes under low-wind conditions and emphasize the need for experimentally constrained parametrizations tailored to freshwater environments. By providing online measurements with minimal sampling losses of ammonia and alkylamine water-to-air exchange, this work advances our understanding of the physicochemical controls governing their emission dynamics and supports improved representation of aquatic nitrogen sources in atmospheric models.

## Supplementary Material



## Data Availability

The data that support the findings of this study will be posted and available on KiltHub upon publication of the manuscript.

## Notes

CIMS signal-to-concentration conversion, zero air and nitrogen gas background measurements, Two-Film Theory equations, Henry’s Law solubility constants and p*K*_a_ values, Two-Film Theory and CIMS flux comparisons, and effects of varying aqueous volumes and bubbling (PDF)
