# Supplementary material for: Behavior of Water-to-Air Trace Gas Exchange for Ammonia and Amines at Low Wind Speeds Using Chemical Ionization Mass Spectrometry
Source: ACS Earth Space Chem. 2026 Jul 30;10(8):1843–54. doi: 10.1021/acsearthspacechem.6c00080 (PMC13501951; doi:10.1021/acsearthspacechem.6c00080)
Supplement: Supplementary file 1 [file sp6c00080_si_001.pdf]

Supplement of

Behavior of Water-to-Air Trace Gas Exchange for Ammonia and Amines at Low Wind Speeds Using Chemical Ionization Mass Spectrometry

Christine Troller[1,2], Stephen D. Archer,[3] Coty N. Jen[1,2*]

[1] Department of Chemical Engineering, Carnegie Mellon University, 5000 Forbes Ave, Pittsburgh, PA 15213, USA

[2] Center for Atmospheric Particle Studies, Carnegie Mellon University, 5000 Forbes Ave, Pittsburgh, PA 15213, USA

[3] Bigelow Laboratory for Ocean Sciences, 60 Bigelow Drive, East Boothbay, ME 04544, USA

*Email: cotyj@andrew.cmu.edu

Pages S1-S9. Figures S1-S4. Table S1

Contents

S1. CIMS Ion Signal to Gas Concentration Conversion

S2. Gas Concentrations Measured from Zero Air and Nitrogen Gas

S3. Two-Film Resistance Theory Equations and Parameters

S4. Henry's Law Solubility Constants and pKa values

S5. Two-Film Resistance Theory Comparison to CIMS Fluxes

S6. Effect of Varying Aqueous Solution Volumes

S7. Effect of Bubbling the Aqueous Solution with Zero Air and Nitrogen Gas

S1. CIMS Ion Signal to Gas Concentration Conversion

The measured ion signal (Hz) for each mass detected in the sample flow is converted to the gaseous species concentration by assuming the following elementary chemical ionization reaction: $X + (H_2O)_n \bullet H_3O^+ \rightarrow HX^+ + (H_2O)_n$. This reaction occurs at the ion-molecule collision limit, k, which is taken to be 2 x 10^{-9} cm^3 s^{-1}.(1,2)

The reagent ion concentration is assumed to be high and thus does not change with time. Previous studies have shown that the gas concentration of analyte ion ([X]) does not change by more than 5% during the short chemical ionization processor.(3)

The CIMS signal is then directly proportional to gas concentration and the chemical ionization reaction time (t_{CI}). The chemical ionization time is determined from the geometry and electric field of the CIMS inlet. For the transverse inlet of the CIMS, the ions are assumed to travel in a parallel plate electric field. As a result, t_{CI} is determined from the plate distance, which refers to the distance between the front and back of the inlet where decreasing voltages are being applied, the ion mobility, and the electric field. The following equations are used to convert the analyte ion signal, S, to analyte concentration [X]:

$$t_{CI}\ [s] = \frac{plate\ distance\ (cm)}{ion\ mobility\ \left(\frac{cm^2}{V * s}\right) x\ E\ \left(\frac{V}{cm}\right)} \qquad (S1)$$

$$[\boldsymbol{X}]\ [molecules\ cm^{-3}] = \frac{S_X}{S_{19} + S_{37} + S_{55} + S_{73}} MDTE \left(\frac{1}{kt_{CI}}\right) \qquad (S2)$$

Equation S2 contains a ratio of different signals, where S_x is the signal for the analyte, S_{19} is the signal for H_3O^+, S_{37} is the signal for $H_2O \bullet H_3O^+$, S_{55} is the signal for $(H_2O)_2 \bullet H_3O^+$, S_{73} is the signal for $(H_2O)_3 \bullet H_3O^+$.

Mass-dependent transmission efficiency (MDTE) refers to the variation in how effectively a mass spectrometer transmits ions of different mass-to-charge ratios to the detector, which introduces bias into concentration calculations if ions of differing masses are not transmitted equally.(4)

Accounting for MDTE is important when comparing signals across a wide mass range. Thus, signals for all masses observed in this study are corrected from their respective MDTE, values extrapolated from measured MDTEs on a similar instrument as shown in Heinritzi et al.(4)

S2. Gas Concentrations Measured from Zero Air and Nitrogen Gas

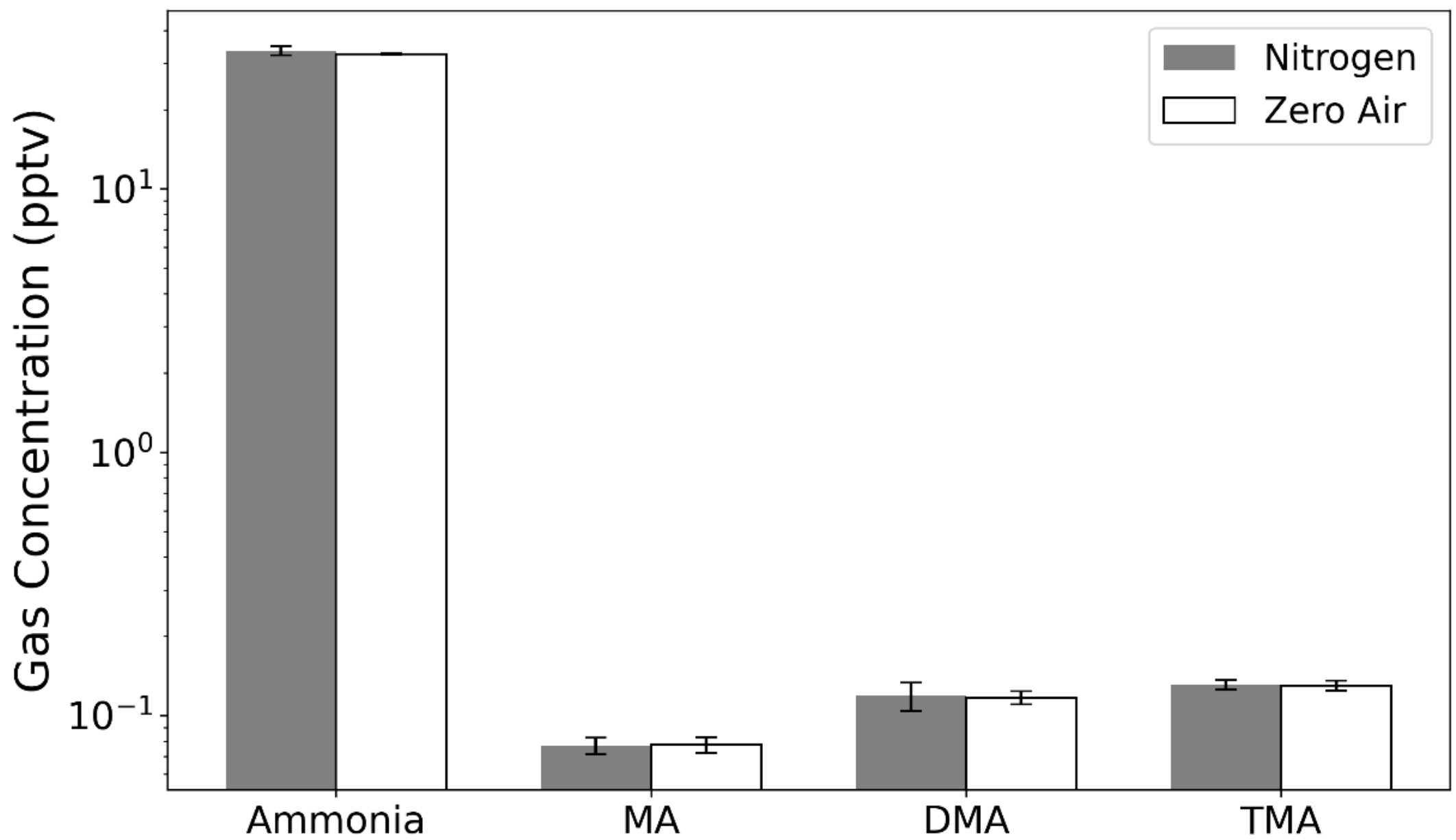

Figure S1. *Nitrogen gas and zero air carrier gas background levels of ammonia and alkylamines, with vertical error bars showing the standard deviation.*

Figure S1 displays average gas concentrations measured from the empty FMS when either 5 L min^{-1} of pure nitrogen or zero air is flowed through the tank headspace for 3 hrs. This comparison helps assess whether zero air introduces ammonia or alkylamines into the system. Pure nitrogen contains extremely minimal amounts (below detection limits) of ammonia or alkylamines as measured by the CIMS without the FMS (not shown). Both carrier gases showed similarly low background levels, confirming that zero air does not contribute additional ammonia or amines to the measurements.

S3. Two-Film Resistance Theory Equations and Parameters

The overall air-water gas transfer velocity (k_t) is calculated using the following equation:

$$\frac{1}{k_t} = \frac{1}{k_w} + \frac{1}{Hk_a} \qquad (S3)$$

where k_w and k_a are liquid and air mass transfer coefficients, respectively. H is the dimensionless gas-over-liquid Henry's Law constant, converted from Henry's Law values (H_s^{cp}) in **Table S1** using the following equation:

$$H = \frac{1}{H_s^{cp}RT} \qquad (S4)$$

where R is the gas constant (0.082 L atm^{-1} mol^{-1} K^{-1}) and T is 298.15 K. Since ammonia and alkylamines are water-soluble, their water-side resistance (k_w) is negligible, making the overall mass transfer effectively governed by the air-side resistance ($k_t \approx Hk_a$). The air-side mass transfer coefficient k_a can be calculated using the following equation:

$$k_a(m\,s^{-1}) = 0.001 + \frac{u_*}{13.3\sqrt{Sc_a} + C_d^{-1/2} - 5 + \frac{\ln(Sc_a)}{2\kappa}} \qquad (S5)$$

where $u_*$is the friction velocity, C_d is the drag coefficient, and Sc_a is the Schmidt number for air, and are calculated from equations given in Johnson (2010).(5) The von Karman constant, κ, is taken to be 0.4.(5–7)

S4. Henry's Law Solubility Constants and pKa values

Table S1. *Henry's Law solubility constants (H_s^{cp}) and pKa values with superscripts indicating the reference for that value.*

Compound	pKa (25°C)	H_s^{cp} lower limit (M/atm)	H_s^{cp} upper limit (M/atm)
Ammonia	9.25[8]	27[9]	77[10]
Methylamine	10.62[11]	35[12]	120[13]
Dimethylamine	10.73[14]	30[12]	180[15]
Trimethylamine	9.8[16]	3.3[17]	130[15]

Table S1 lists the pKa values and the lower and upper bounds of Henry's law constants (Hscp) used to estimate the gas-phase concentrations of ammonia and amines based on Henry's Law, as shown in **Figures 4–7**. These parameters are critical for modeling the partitioning behavior of each compound between the aqueous and gas phases.

S5. Two-Film Resistance Theory Comparison to CIMS Fluxes

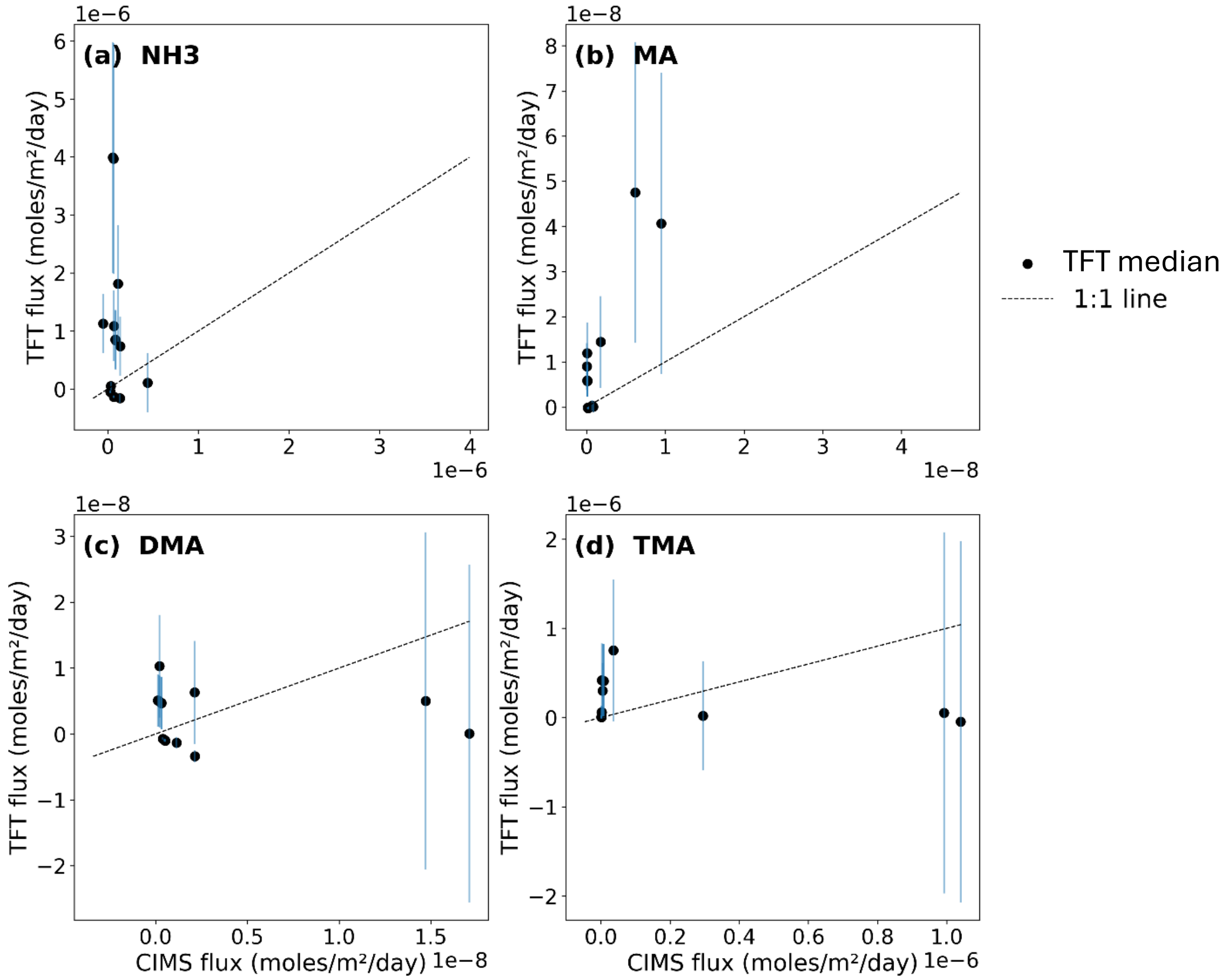

Figure S2. *Overprediction of fluxes from the two-film resistance theory (TFT) compared to observed CIMS fluxes for (a) ammonia (NH3), (b) methylamine (MA), (c) dimethylamine (DMA), and (d) trimethylamine (TMA). Blue vertical lines represent the range of TFT fluxes predicted with the range of Henry's Law constants used in this work.*

Figure S2 compares the observed CIMS fluxes with the calculated fluxes predicted by the two-film resistance model for ammonia and alkylamines. The model typically either predicts the wrong flux direction or overestimates the flux by orders of magnitude. This suggests that the parameters governing the TFT are likely not the primary factors controlling the fluxes of these compounds under the very low-wind conditions in the FMS.

S6. Effect of Varying Aqueous Solution Volumes

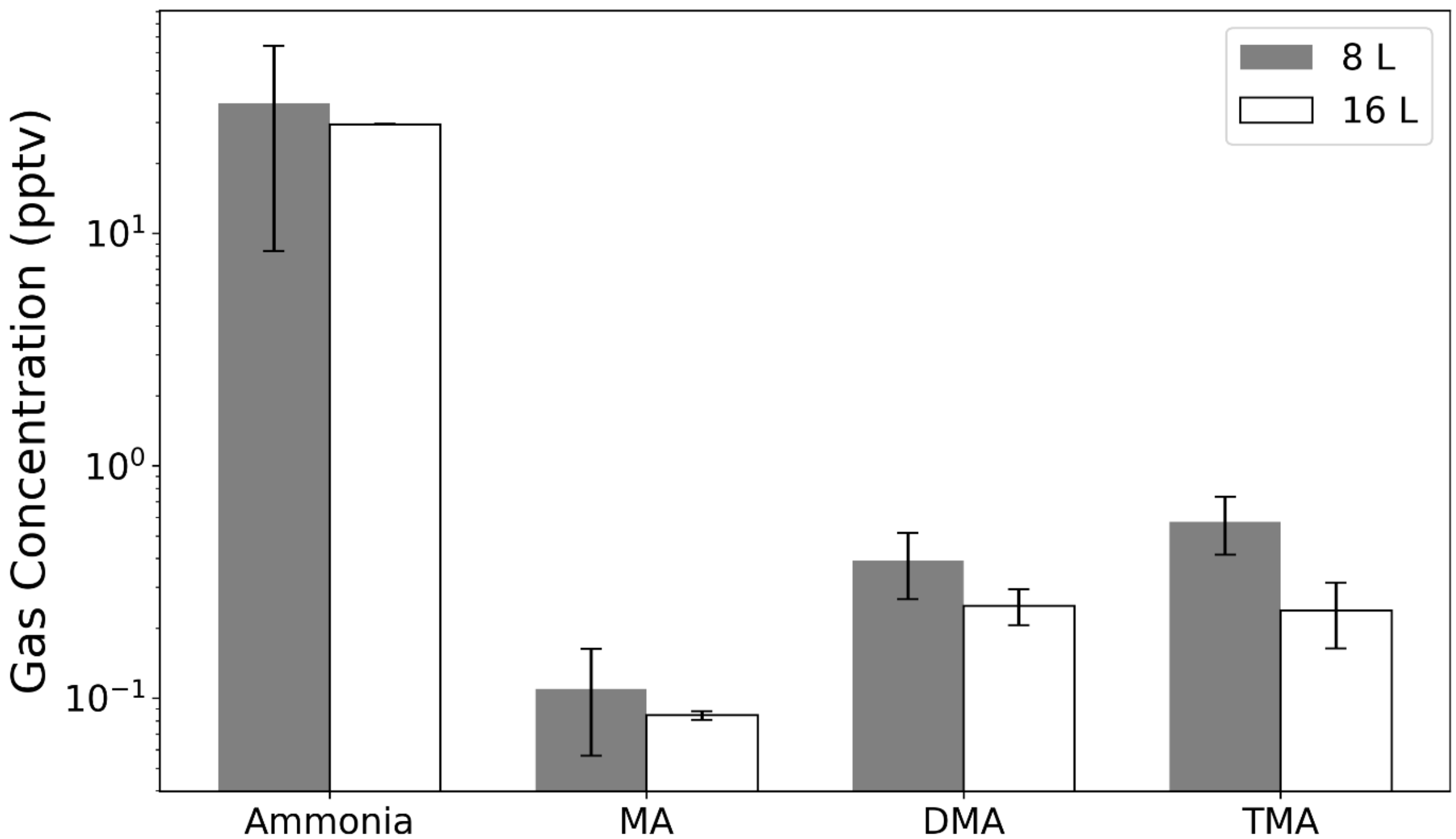

Figure S3. *Gas concentrations measured from 8 L and 16 L identical concentration aqueous solutions. Error bars are the standard deviation of the experiments.*

Figure S3 displays the 5 hr average gas-phase concentrations of ammonia and alkylamines from duplicate experiments using identical aqueous concentrations but different solution volumes (8 L vs. 16 L). Overall, the 16 L solutions produced slightly lower, however still similar, gas-phase concentrations compared to the 8 L tests. This difference may be attributed to a longer equilibration time required for the larger volume to saturate the tank headspace. However, the observed variation is small and falls within the range of experimental uncertainty.

S7. Effect of Bubbling the Aqueous Solution with Zero Air and Nitrogen Gas

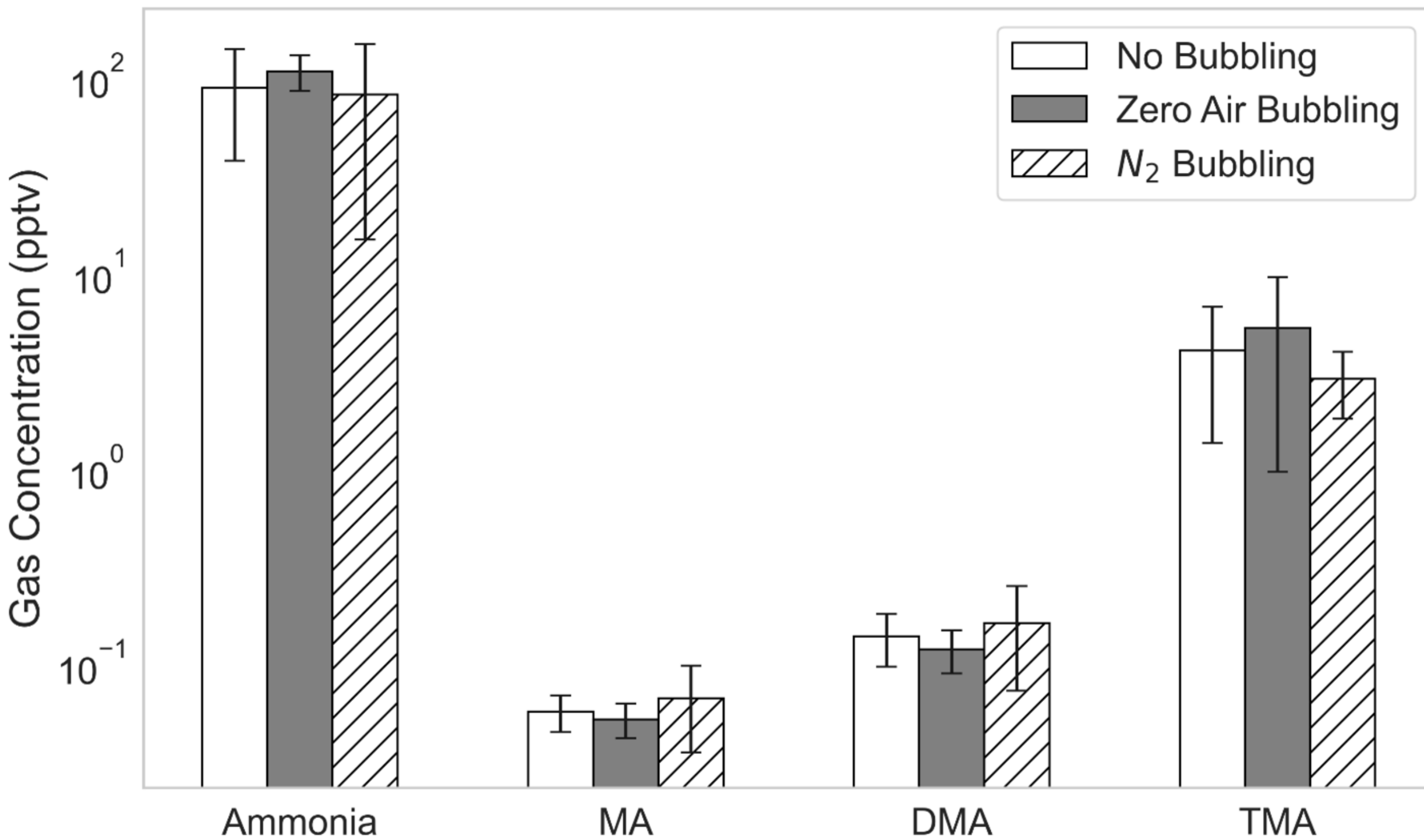

Figure S4. *2 hr average gas concentrations of compounds under non-bubbling (white), 280 cm^3 min^{-1} zero air bubbling (gray), and 280 cm^3 min^{-1} N_2 bubbling (striped) conditions. Error bars show the standard deviation over the duplicate trials.*

Figure S4 displays the 2 hr average gas-phase concentrations of ammonia, MA, DMA, and TMA under three conditions: no bubbling, bubbling with 280 cm^3 min^{-1} zero air, and bubbling with 280 cm^3 min^{-1} nitrogen (N_2). The bubbling mechanism consisted of 1/8" Teflon tubing, with perforations along the final 8" segment of the tube and a stainless-steel Swagelok cap affixed to the end to ensure the bubbler remained submerged at the bottom of the tank. The introduction of bubbling, whether with zero air or nitrogen, did not result in significant changes in the gas-phase concentrations of the tested compounds.

This lack of response to bubbling further suggests that the FMS system was already near gas–liquid equilibrium during the experiments. If equilibrium had not yet been reached, enhanced mixing and surface renewal from bubbling would be expected to increase volatilization and increase the observed gas phase concentrations. It is likely that the compounds' volatilization rates are more strongly governed by thermodynamic limits (e.g., Henry's Law partitioning) rather than gas-phase transport resistance, or that the specific bubbling configuration did not significantly enhance interfacial transfer due to bubble size or flow regime.

References

(1) Španěl, P.; Smith, D. Selected Ion Flow Tube Studies of the Reactions of H3O+, NO+, and O2+ with Several Amines and Some Other Nitrogen-Containing Molecules. *Int. J. Mass Spectrom.* **1998**, *176* (3), 203–211. https://doi.org/10.1016/S1387-3806(98)14031-9.

(2) Su, Timothy.; Bowers, M. T. Ion-Polar Molecule Collisions. Effect of Molecular Size on Ion-Polar Molecule Rate Constants. *J. Am. Chem. Soc.* **1973**, *95* (23), 7609–7610. https://doi.org/10.1021/ja00804a011.

(3) Berresheim, H.; Elste, T.; Plass-Dülmer, C.; Eisele, F. L.; Tanner, D. J. Chemical Ionization Mass Spectrometer for Long-Term Measurements of Atmospheric OH and H2SO4. International Journal of Mass Spectrometry **2000**, 202, 91–109. https://doi.org/10.1016/S1387-3806(00)00233-5

(4) Heinritzi, M.; Simon, M.; Steiner, G.; Wagner, A. C.; Kürten, A.; Hansel, A.; Curtius, J. Characterization of the Mass-Dependent Transmission \hack\newline Efficiency of a CIMS. *Atmospheric Meas. Tech.* **2016**, *9* (4), 1449–1460. https://doi.org/10.5194/amt-9-1449-2016.

(5) Johnson, M. T. A Numerical Scheme to Calculate Temperature and Salinity Dependent Air-Water Transfer Velocities for Any Gas. *Ocean Sci.* **2010**, *6* (4), 913–932. https://doi.org/10.5194/os-6-913-2010.

(6) Crusius, J.; Wanninkhof, R. Gas Transfer Velocities Measured at Low Wind Speed over a Lake. *Limnol. Oceanogr.* **2003**, *48* (3), 1010–1017. https://doi.org/10.4319/lo.2003.48.3.1010.

(7) Carpenter, L. J.; Archer, S. D.; Beale, R. Ocean-Atmosphere Trace Gas Exchange. *Chem Soc Rev* **2012**, *41* (19), 6473–6506. https://doi.org/10.1039/C2CS35121H.

(8) Haynes, W. M., Ed. CRC Handbook of Chemistry and Physics, 95th Ed.; CRC Press: Boca Raton, FL, **2014**; p 5-92.

(9) Dean, J. A.; Lange, N. A. Lange's Handbook of Chemistry, 15th Ed.; McGraw-Hill: New York, **1999**; ISBN 9780070163843.

(10) Holzwarth, G.; Balmer, R. G.; Soni, L. The Fate of Chlorine and Chloramines in Cooling Towers. Water Res. **1984**, 18, 1421–1427. https://doi.org/10.1016/0043-1354(84)90012-5.

(11) Dean, J. A. Handbook of Organic Chemistry; McGraw-Hill Book Co.: New York, NY, **1987**; p 8–38.

(12) Wilhelm, E.; Battino, R.; Wilcock, R. J. Low-Pressure Solubility of Gases in Liquid Water. Chem. Rev. **1977**, 77, 219–262. Https://Doi.Org/10.1021/Cr60306a003.

(13) Hilal, S. H.; Ayyampalayam, S. N.; Carreira, L. A. Air–Liquid Partition Coefficient for a Diverse Set of Organic Compounds: Henry's Law Constant in Water and Hexadecane. Environ. Sci. Technol. **2008**, 42, 9231–9236. https://doi.org/10.1021/Es8005783.

(14) Perrin, D. D. Dissociation Constants of Organic Bases in Aqueous Solution; IUPAC Chemical Data Series; Butterworths: London, **1965**.

(15) Duchowicz, P. R.; Aranda, J. F.; Bacelo, D. E.; Fioressi, S. E. QSPR Study of the Henry's Law Constant for Heterogeneous Compounds. Chem. Eng. Res. Des. **2020**, 154, 115–121. https://doi.org/10.1016/j.Cherd.2019.12.009.

(16) Perrin, D. D. Dissociation Constants of Organic Bases in Aqueous Solution, Suppl. 1972; IUPAC Chemical Data Series; Butterworths: London, **1972**.

(17) Modarresi, H.; Modarress, H.; Dearden, J. C. QSPR Model of Henry's Law Constant for a Diverse Set of Organic Chemicals Based on Genetic Algorithm–Radial Basis Function Network Approach. Chemosphere **2007**, 66, 2067–2076. https://doi.org/10.1016/j.Chemosphere.2006.09.049.
